# Supplementary material for: Identification of Risk Factors Associated with Resistant Escherichia coli Isolates from Poultry Farms in the East Coast of Peninsular Malaysia: A Cross Sectional Study
Source: Antibiotics (Basel). 2021 Jan 26;10(2):117. doi: 10.3390/antibiotics10020117 (PMC7912622; doi:10.3390/antibiotics10020117)
Supplement: Supplementary file 1 [file antibiotics-10-00117-s001.pdf]

Tale S1: Characteristics of 31 farmers in Kelantan, Terengganu and Pahang states, Malaysia

Variables	Household farms
Age of farm manager (years), median (IQR)	45(39-54)
Gender	
Female	5(16.1)
Male	26 (83.9)
Education	
Primary	9(29.0)
Secondary	14(45.2)
College	8(25.8)
Use of antibiotics	
Yes	31 (100)
No	0
Vaccines	
Yes	31(100)
No	0
Recent disease history	
Yes	22(71.0)
No	9(29.0)
Animal contact	
Yes	2 (6.5)
No	29(93.5)
Production system	
Broiler	16(51.6)
Layer	5(16.1)
Mixed	10 (32.3)
Management system	
Intensive	16(51.6)
Semi-intensive	12 (38.7)
Mixed	3(9.7)
Farm size	
Small	10(32.3)
Medium	13(41.9)
Large	8(25.8)
Flock origin	
Local	3(9.7)
Imported	9(29.0)
Both	19(61.3)
States	
Kelantan	13(41.9)
Terengganu	7(22.6)
Pahang	11(35.5)
Districts	
Bachok	2(6.5)
Kota Bharu	3(9.7)
Machang	1(3.2)
Pasir Mas	2(6.5)
Pasir Puteh	2(6.5)
Jeli	3(9.7)
Kuantan	6(19.4)
Maran	1(3.2)

Pekan	4(12.9)
Kuala terengganu	3(9.7)
Marang	4(12.9)
Sewage system	
Excellent	11(35.5)
Good	17(54.8)
Poor	3(9.7)
Visitors	
Yes	19(61.3)
No	12(38.7)
PPE	
Yes	19(61.3)
No	12(38.7)
Washing facilities	
Yes	25(80.6)
No	6(19.4)
Use of disinfectant	
Yes	21(67.7)
No	10(32.3)
Source of antibiotic	
Drug supplier	20(64.5)
Feed store	11(35.5)
Veterinary services	
Yes	31(100)
No	0
Source of feed	
Endogenous	12(38.7)
Exogenous	16(51.6)
Water source	
Surface water	6(19.4)
Bond water	13(41.9)
Pump water	12 (38.7)
